# Fulminant Amebic Colitis after Corticosteroid Therapy: A Systematic Review

**DOI:** 10.1371/journal.pntd.0004879

**Published:** 2016-07-28

**Authors:** Debbie-Ann Shirley, Shannon Moonah

**Affiliations:** 1 Department of Pediatrics, University of Virginia School of Medicine, Charlottesville, Virginia, United States of America; 2 Department of Medicine, University of Virginia School of Medicine, Charlottesville, Virginia, United States of America; CINVESTAV, MEXICO

## Abstract

**Background:**

Amebic colitis, caused by intestinal infection with the parasite, *Entamoeba histolytica*, is a common cause of diarrhea worldwide. Fulminant amebic colitis is the most devastating complication of this infection, associated with both high mortality and morbidity. We conducted a review of the English literature to describe cases of fulminant amebic colitis associated with exposure to corticosteroid medications in order to identify the risk factors for poor outcome and determine difficulties in diagnosis and treatment.

**Methodology and Principal Findings:**

Articles reporting severe and fulminant forms of amebic colitis between 1991 and 2016 were collected. 525 records were screened to identify 24 cases for qualitative analysis associated with corticosteroid use. Cases arose from areas of high endemicity or travel to such areas. Most cases (14 of 24, 58%) were given corticosteroids for initially misdiagnosed colitis, mainly inflammatory bowel, resulting in rapid progression of disease. Nearly half of all cases underwent surgical intervention, and 25% of cases died, despite all patients eventually receiving treatment with metronidazole. The odds of death did not differ significantly by prior misdiagnosis, co-morbidities, bowel perforation or need for surgery.

**Conclusions and Significance:**

Infection with *E*. *histolytica* should be considered prior to the administration of corticosteroids, in particular for patients residing in endemic areas or those with appropriate travel history, especially prior to the diagnosis of inflammatory bowel disease. The development of preventative and treatment interventions are needed to improve outcomes of fulminant disease.

## Author Summary

*Entamoeba histolytica* is one of the most important enteric pathogens affecting people worldwide, causing the diarrheal disease amebic colitis. Fulminant amebic colitis is an uncommon but life threatening complication that may ensue. High rates of colonization with *E*. *histolytica* burden many developing countries, and travelers are at risk of acquisition of infection when they visit endemic areas. Corticosteroids are an invaluable group of broadly prescribed anti-inflammatory medications, but have been identified as a risk factor for the development of fulminant amebic colitis. Our comprehensive report highlights the frequent misdiagnosis of amebic colitis and the high morbidity and mortality associated with fulminant disease. Improved awareness of this condition among medical providers is needed, so that infection with *E*. *histolytica* can be considered in both patients presenting with symptoms of colitis and patients with asymptomatic colonization prior to the administration of corticosteroids. This study points to the need to continue efforts to develop both a vaccine that can prevent amebic colitis and innovative life- and bowel-saving adjuncts for the treatment of fulminant amebic colitis through an improved understanding of host responses to infection with *E*. *histolytica*.

## Introduction

Amebic colitis, caused by the protozoan parasite *Entamoeba histolytica*, is a leading cause of severe diarrhea worldwide, killing more than 55,000 people globally each year [1]. The greatest burden of amebic disease occurs in developing countries, likely due to poorer socioeconomic conditions, decreased sanitation and reduced hygiene [2]. In the largest global study of childhood diarrheal illness conducted to date, *E*. *histolytica* was shown to be a top cause of severe diarrhea among infants and children living in Africa and Asia, and was the leading cause of unadjusted mortality in the 12 to 24 month age group [3]. The parasite has a two-stage life cycle, existing as either an infectious cyst or invasive trophozoite. Transmission occurs via ingestion of cysts, most commonly by fecally contaminated food or water, though direct fecal-oral transmission through sexual contact is also described [2,4]. Trophozoite invasion of the intestinal mucosa leading to mucosal inflammation is a hallmark of amebic colitis.

The vast majority of amebic infections are asymptomatic, with approximately 10% of those infected progressing to have symptoms. Amebic colitis is the most common symptomatic manifestation, with variable presentation, including watery diarrhea, dysentery, abdominal pain, tenderness and rarely the formation of a tumor like granulation mass referred to as an ameboma [2,5]. Trophozoites can disseminate to the liver, causing amebic abscesses, as well as to the central nervous system and other extra-intestinal sites. Fulminant amebic colitis, though uncommon, is the most serious and life-threatening complication of amebiasis, presenting initially with bloody diarrhea, fever, leukocytosis and abdominal pain. Bowel necrosis, toxic megacolon, perforation and peritonitis may ensue. Fulminant amebic colitis is associated with high mortality and morbidity, with case fatality rates ranging from 40% to 89% [6–10].

Corticosteroids are commonly prescribed in medicine for their anti-inflammatory and immunosuppressive therapeutic properties. Case reports have indicated that corticosteroids are a risk factor predisposing to the development of fulminant amebic colitis [11–13]. Given the widespread use of corticosteroids, this observation holds significant implications not only to those living in endemic areas, but with expanding patterns in travel and migration, also poses an emerging health threat to those living in more industrialized settings. Our aim was to systematically review recent articles reporting fulminant amebic colitis in patients treated with corticosteroids to identify the main risk factors for poor outcome and highlight challenges regarding diagnosis and treatment.

## Methods

We carried out a systematic review of articles published in the English literature between January 1991 and May 2016. The search was performed electronically in PubMed to find all articles reporting amebic colitis, using the following strategy: disease ("amoebic colitis"[All Fields] OR "dysentery, amebic"[MeSH Terms] OR ("dysentery"[All Fields] AND "amebic"[All Fields]) OR "amebic dysentery"[All Fields] OR ("amebic"[All Fields] AND "colitis"[All Fields]) OR "amebic colitis"[All Fields]) AND (("199101/01"[PDAT]: "2016/05/01"[PDAT]) AND "humans"[MeSH Terms]). The search was last conducted on May 5, 2016. A similar search was performed using Google Scholar to find additional articles that may not have been cataloged in PubMed. Case reports of intestinal amebiasis were then then reviewed to determine corticosteroid exposure, including administration of systemic preparations (prednisone, prednisolone, methylprednisolone, triamcinolone, dexamethasone, cortisone acetate and hydrocortisone) as well as enema preparations (budesonide). Evidence of amebiasis included identification by stool study, tissue examination or serology. Fulminant colitis was defined as severe abdominal pain, dysentery, fever, peritonitis, perforation or the need for urgent surgical intervention [14]. When possible, odds ratios were calculated to measure association between exposure and outcome. A two-tailed p-value of <0.05 was considered to be statistically significant and was determined by use of STATA, version 11 (StataCorp).

## Results

**Fig 1:**
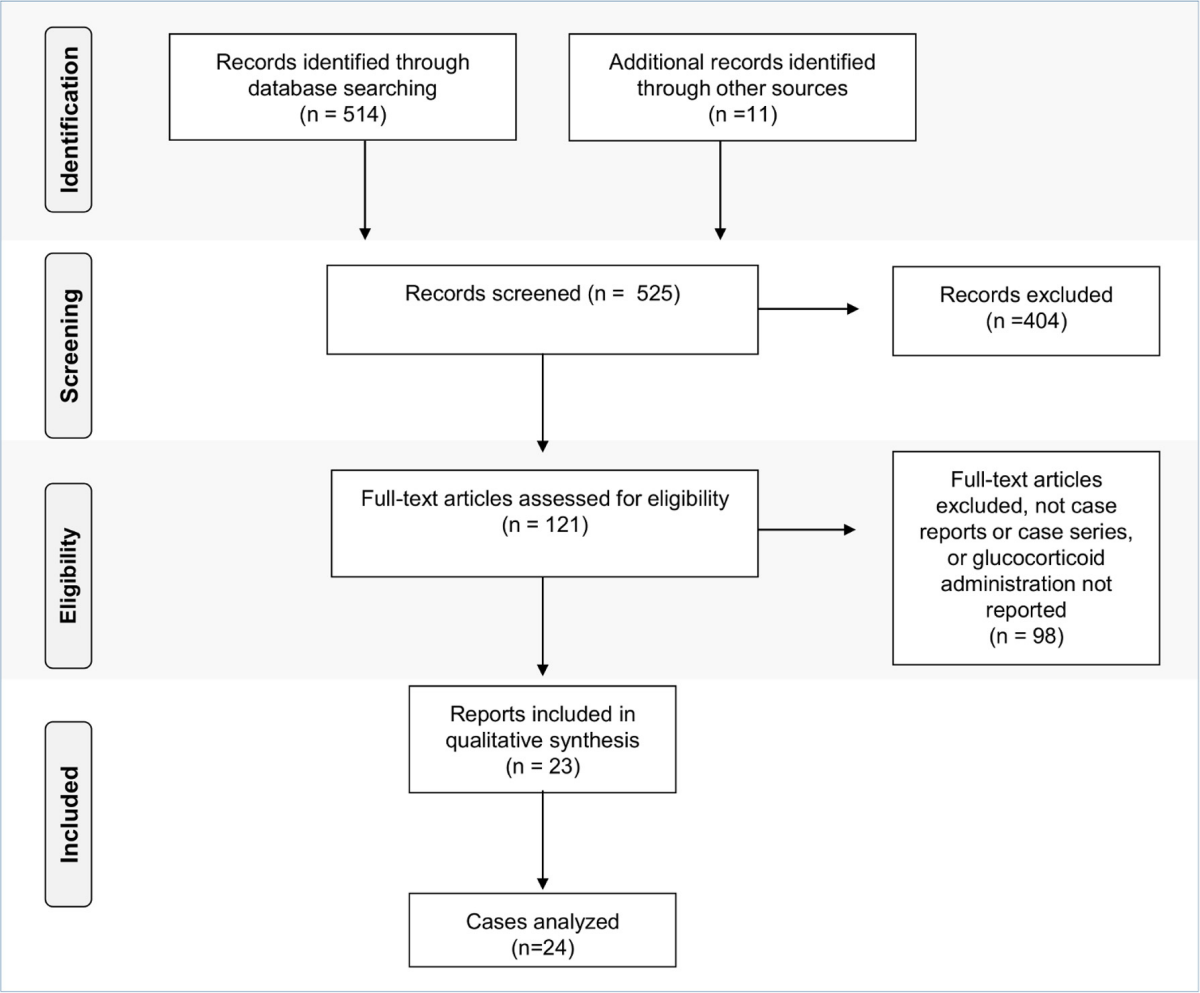
PRISMA flow chart: Data collection and selection of case studies.

Our search strategy identified 514 publications. Eleven additional publications were identified using the alternative search engine, excluding duplicate publications. By evaluation of the title and abstract, 404 publications were excluded. Full text publications were then reviewed for eligibility including reports of intestinal amebiasis and concomitant administration of corticosteroid therapy. Among the 122 publications reviewed, a total of 23 publications, with 24 cases were found in the literature (**Fig 1**).

### Demographics

Cases were reported from many regions of the world, including those with both high endemicity and non-endemicity of amebiasis (**Table 1**). The majority of reports arose from Asia (11 of 24 cases, 46%) including Japan [15–19], Taiwan [20,21], India [22], Singapore [23], Pakistan [24] and South Korea [25]. This was followed by European countries (6 of 24 cases, 25%), including Spain [26,27], Sweden [28], Denmark [29], Portugal [30] and Italy [31]. There were three reports (12%) originating from the USA [32–34] and a fourth (4%) from the USA territory American Samoa [35]. Two cases (8%) from a single case series were from Chile [36]. There was a single case (4%) from Israel, representing the Middle East [37]. There were no reports from Africa, Central America or the Caribbean. Many cases arising from countries with low or no endemicity postulated travel to endemic countries as the most likely risk factor for transmission of infection. Reports arising from European countries reported travel to Bali [29], the Philippines [26], Brazil, Saudi Arabia, Sri Lanka, Tanzania [28], Angola [30], the Far East and North Africa [27]. Travel history of the partner was noted to be the most likely source for one case [27]. Among reports arising from the USA, travel to Mexico was listed as a significant risk factor [33]. Another report arising from the USA did not report travel history, but contaminated well water was suspected to be the source for transmission of infection [34]. Cases aged in range from 1–83 years with a median age of 41 years (**Table 1**), including three pediatric cases [35,36].

**Table 1:** Clinical features and outcomes of cases with severe or fulminant amebic colitis following treatment with corticosteroids, 1991–2016.

Age (y)	Primary steroid indication	Co-morbid condition	Steroid form	Other immune modulating Rx	Country reported	Risk factor acquisition of amebiasis	Symptoms of amebic colitis	Area of gut involved	MTZ Rx	Surgery	Survival	Ref
**60**	Misdiagnosed UC	-	E	Mesa	Japan	-	A, WL, D, H	R	Y	N	Y	[15]
**57**	GVHD	BMT for AML	PO	FK508, ATG, MTX	Japan	-	AP, D, H, T	R, DC, TC	Y	N	Y	[16]
**60**	Multiple myeloma	CMV colitis	PO	-	Japan	Travel to endemic country*	D, H, F	NS	Y	N	N	[17]
**48**	Misdiagnosed UC	-	PO	Mesa	Japan	-	P	PC	Y	Y	Y	[18]
**55**	Misdiagnosed UC	-	IV	5-ASA	Japan	-	D, H, AP. TM	NS	Y	Y	N	[19]
**50**	Nephrotic syndrome	Misdiagnosed UC; HIV; CMV/ fungal colitis	PO, IV	5-ASA, Mesa	Taiwan	-	H, P	PC	Y	Y	N	[20]
**34**	Interstitial pneumonia	AIDS; CMV colitis; MAC pneumonitis	NS	-	Taiwan	-	AP, D, P	C	Y	Y	Y	[21]
**68**	Misdiagnosed IBD	Alcoholism	PO	5-ASA	India	Endemic	D, H, AP, P	PC	Y	Y	N	[22]
**31**	Misdiagnosed lupus of the gut	Lupus; Salmonellosis	PO, IV	-	Singapore	Endemic	D, H, AP, F	PC	Y	N	N	[23]
**35**	Misdiagnosed abdominal TB	Hepatitis C infection	PO	-	Pakistan	Endemic	AP, D, F	IC, Am	Y	Y	Y	[24]
**47**	Misdiagnosed intestinal vasculitis	Lupus	PO, IV	Hydroxy-chloroquine	South Korea	-	AP, H, P	PC	Y	Y	Y	[25]
**28**	Misdiagnosed UC	-	PO	-	Spain	Travel to Philippines	D, H, F, WL	PC	Y	N	Y	[26]
**33**	Thrombotic thrombocytopenic purpura	Transfusion related acute lung injury	Sys	Plasmapheresis	Spain	Partner travelled to the Far East and North Africa	AP, D, H, P	C	Y	Y	Y	[27]
**41**	Misdiagnosed IBD	-	E, IV	5-ASA	Sweden	Travel to Brazil, Saudi Arabia, Sri Lanka, Tanzania	D, AP	R, DC, TC	Y	N	Y	[28]
**66**	Misdiagnosed IBD	HTN; psoriatic arthritis	PO, IV	MTX	Denmark	Travel to Bali	AP, D, H, WL	AC, TC, Am	Y	N	Y	[29]
**39**	Misdiagnosed CD	-	PO	-	Portugal	Travel to Angola	H, A, WL, AP, F	AC, C, LA	Y	N	Y	[30]
**36**	GVHD	BMT for AML; CONS sepsis	PO	MXT, CSa	Italy	Travel to North Africa	AP, D, H	TC, C, PS	Y	N	Y	[31]
**83**	Pneumonia	-	PO	-	USA	-	H, F	AC, LA	Y	N	Y	[32]
**56**	Misdiagnosed UC	-	IV, PO	5-ASA, Mesa	USA	Travel to Mexico	D, H, AP, WL, P	PC, LA	Y	Y	Y	[33]
**42**	Liver transplant	Hepatitis C cirrhosis	PO	FK508, AZA	USA	Well water	AP, D, T	AC, TC	Y	N	Y	[34]
**1**	Bronchiolitis	*Streptococcus pyogenes* bacteremia*	IV	-	American Samoa	Polynesian	D, P	Jejunum	Y	Y	Y	[35]
**4**	GVHD	BMT for AML; GNS	PO	CSa	Chile	Endemic	D, AP, F	R	Y	N	Y	[36]
**15**	GVHD	BMT for AML; CMV and fungal colitis	PO, IV	CSa	Chile	Endemic	D, AP, F	PC	Y	N	N	[36]
**37**	CD	-	IV, PO	5-ASA, 6-MP	Israel	-	F, H, D	AC	Y	Y	Y	[37]

* Contact with sex workers; *A*, anorexia; *AC*, Ascending colon; *Am*, ameboma; *AML*, acute myeloid leukemia; *AP*, abdominal pain; *ATG*, antithymocyte globulin; *AZA*, azathioprine; *BMT*, bone marrow transplant; *CD*, Crohn disease; *CMV*, cytomegalovirus; CONS, coagulase-negative *Staphylococcus aureus*; *CSa*, cyclosporine; *D*, diarrhea; *DC*, descending colon; *E*, enema; *F*, fever; *FK508*, tacrolimus; *GNS*; Gram negative sepsis; *GVHD*, graft-versus-host disease; *H*, bloody stools; *IBD*, inflammatory bowel disease; *IC*, ileocolic; *IV*, intravenous; *LA*, liver abscess; *ME*, meningoencephalitis; *Mesa*, mesalamine; *MTX*, methotrexate; MTZ, metronidazole; *N*, no; *NS*, not stated; *P*, perforation; *PC*, pancolitis; *PO*, by mouth; *PS*; proctosigmoid; *R*, rectum; *Rx*, treatment; *T*, tenesmus; *TB*, tuberculosis; *TC*, transverse colon; *TM*, toxic megacolon; *UC*, ulcerative colitis; *WL*, weight loss; *Y*, yes; *5-ASA*, sulfasalazine; *6-MP*, mercaptopurine

### Indications for corticosteroid use

In all, 14 of 24 (58%) cases were given corticosteroid medications for initially misdiagnosed colitis (**Table 1**), including misdiagnosed inflammatory bowel disease (11 of 24 cases, 46%) [18–20,22,26,28–30,33,37], intestinal vasculitis (2 of 24 cases, 8%) [23,25], and abdominal tuberculosis (1 of 24 cases, 4%) [24]. The remainder were given corticosteroid medications for the indication of prevention or treatment of graft-versus-host disease (GVHD) following transplantation (5 of 24, 21%) [16,31,34,36], respiratory illness (3 of 24 cases, 12.5%) [21,32,35], multiple myeloma (1 of 24, 4%) [17] and thrombocytopenic purpura (1 of 24, 4%) [27].

Co-morbidities in this series were high (**Table 1**) and included 4 cases (17%) with underlying autoimmune disease [23,25,27,29]; 3 cases (12.5%) who underwent either stem cell or solid organ transplantation [16,31,34] and 2 cases (8%) with Human Immunodeficiency virus (HIV) co-infection [20,21]. Histologic evidence of cytomegalovirus (CMV) colitis was present in both of the cases with HIV, as well as a third case without additional immunocompromising state reported [20,21,25]. Eight cases (33%) had no underlying co-morbidity reported. Fourteen cases (58%) were treated with other immune modulating therapies in addition to corticosteroids. These included the anti-inflammatory agents sulfasalazine and mesalamine (8 of 24, 33%) [15,18–20,22,28,33,37], antimetabolites azathioprine or mercaptopurine (2 of 24, 8%) [34,37], methotrexate (3 of 24, 12.5%) [16,29,31], tacrolimus (2 of 22, 8%) [16,34], cyclosporine (3 of 24, 12.5%) [31,36], anti-thymocyte antiglobulin (1 of 24, 4%) [16] and hydroxychloroquine (1 of 24, 4%) [25]. The case with thrombocytopenic purpura underwent plasmapheresis while also receiving corticosteroids and prior to aggravation of symptoms.

The majority of cases were treated with high dose systemic corticosteroids, either enterally or parentally administered, prior to development or worsening of symptoms. Two cases (8%) however, seemed to worsen after treatment with corticosteroid enemas [15,28]. Corticosteroid preparation, dose administered and exact duration of time prior to worsening were inconsistently reported, prohibiting calculation of average cumulative dosages.

### Clinical features

All cases met the study definition of either severe or fulminant amebic colitis and presented with a combination of acute worsening of abdominal pain, diarrhea, and /or bloody stools/ dysentery. Eight (33%) cases were complicated by intestinal perforation [18,20–22,25,27,33,35]. Perforation occurred in both of the cases with HIV, but none of the transplant cases. The odds of perforation did not differ significantly by prior misdiagnosis, CMV co-infection or treatment with additional immune modulating therapies. Associated amebic liver abscesses were found in three (12.5%) cases [30,32,33]. Central nervous system dissemination occurred in one case (4%), a one-year-old child with jejenal perforation, liver abscess and seizures secondary to meningoencephalitis (trophozoites demonstrated by microscopy in the cerebrospinal fluid) [35]. Amebomas were found in two cases (8%) [24,29]. One case (4%) developed toxic megacolon [19]. One case (4%) believed to have both Crohn disease and amebic colitis developed a rectovaginal fistula [37].

Most cases (13 of 24, 54%) had involvement of multiple areas of bowel, including pancolitis in seven cases (29%) [18,20,22,23,25,26,33]. Proctosigmoid involvement was documented in four cases (17%) [15,16,28,31], descending colon in two cases (8%)[16,28], transverse colon in five cases (21%)[16,28,29,31,34], ascending colon in five cases (21%) [29–32,34], cecal/ ileo-cecal in five cases (21%) [21,24,27,30,31], jejenal in one case (4%) [35] and exact location of colitis not specified in two cases (8%) [17,19].

### Laboratory diagnosis

A diagnosis of amebiasis was established in 22 of 24 (92%) cases by microscopy, serology and/ or histology. Post-mortem examination established the diagnosis in one case, revealing *E*. *histolyti*ca in the ulcerated colonic wall [19]. Presumptive diagnosis was made in another case, on the clinical basis of worsening symptoms with anti-mycobacterial therapy and corticosteroids followed by improvement with metronidazole, and supported by suggestive ulcerations on histology. Leukocytosis was reported in 10 of 13 cases (77%). Stool microscopy identified *Entamoeba* cysts and/or trophozoites in 8 of 16 (50%) cases. Methodology used and number of stools submitted was variable, and not consistently reported. Four of the 8 cases with positive microscopy (50%) had at least one other positive study. Serology was positive in 10 of 15 (67%) cases. Of those with positive serology, 7 of 10 cases (70%) had at least one other positive study. The 5 cases with negative serology included both cases who underwent stem cell transplantation [16,31], a case with AIDS [21], a case with Lupus [23] and a case with Crohn disease [37]. The type of serologic assay performed was not consistently reported. Submitted histology from biopsy at colonoscopy showed tissue trophozoites in 7 of 15 (47%) of cases. In cases who underwent surgery, submitted surgical pathology yielded direct evidence of amebiasis in 8 of 9 (89%) of cases. The use of a direct antigen test was reported in a single case [26].

### Outcomes

All cases were reported to have received appropriate therapy with metronidazole. Most cases also reported use of a luminal agent, such as paromomycin, though not always commercially available [16]. One case did not respond to treatment until corticosteroids were withdrawn [36]. Eleven (46%) underwent surgical treatment of their disease (**Table 1**). There were 6 fatalities (25%), half underwent surgical intervention prior to death (**Table 1**). The odds of death did not differ significantly by prior misdiagnosis, transplant status, HIV co-infection, CMV co-infection, perforation, surgery or presence of additional immune modulating therapies.

## Discussion

We identified 24 cases of severe and fulminant amebic colitis treated with corticosteroids over the past 25 years. To our knowledge, this is the largest comprehensive report of such cases. It is interesting that nearly one-third of cases were in returning travelers. Travel to South and South East Asia, Africa, the Middle East and South America were listed as risk factors for European travelers. Travel to Mexico was significant for one US traveler. Data analyzed from the GeoSentinel Surveillance Network, a worldwide network that performs surveillance and monitoring of travel related illnesses, showed that *E*. *histolytica* is the third most frequently isolated pathogen among returning travelers with infectious gastrointestinal disease, accounting for 12.5% of all microbiologically confirmed cases, with an estimated rate of 14.0 per 1000 returned travelers [38]. Rates varied by region of travel and category of traveler, with the highest rates of amebiasis reported in travelers to South Asia, the Middle East and South America, similar to our findings, and among “missionary/ volunteering” travelers [38]. Amebiasis was not considered in any of our reported travelers when they initially presented leading to misdiagnosis. The low incidence of amebiasis in industrialized countries can lead to unfamiliarity with the clinical presentation. Given the significant morbidity and mortality associated with fulminant disease, it is important for providers to enquire about travel history and screen travelers to and migrants from endemic regions for amebiasis, using an appropriate test, such as the fecal antigen test, even if travel occurred in the distant past [13]. Providers should also inquire about the travel history of close household and sexual contacts [4,39]. The utility of this is demonstrated in the case who acquired amebiasis after her partner travelled to the Far East and North Africa [40].

While the incidence of fulminant amebic colitis is likely to be more common in endemic countries given the high frequency of asymptomatic intestinal carriage in many indigenous populations, it is probable that these episodes are not being published in the literature, leading to underrepresentation in our compiled series. Even among cases included from countries with higher endemicity, amebiasis was often not initially suspected. Inflammatory bowel disease was the most frequently misdiagnosed condition at presentation. The diagnostic dilemma here is that many of the symptoms of amebic colitis overlap with symptoms of inflammatory bowel disease. Complicating the matter is that patients with inflammatory bowel disease may also have amebiasis, a considerable diagnostic challenge in endemic countries [41–44]. In countries where amebiasis is endemic, there should be a low threshold for suspecting in patients who present with symptoms of inflammatory bowel disease. All patients with a new diagnosis of inflammatory bowel disease should be screened for amebiasis with a stool study for fecal antigen testing or serum for amebic serology, especially if they reside in or have a history of travel to an endemic area.

It is important to note that while in most patients the cause of colitis was initially misdiagnosed; almost 40% of cases did not have any gastrointestinal symptoms prior to initiation of corticosteroids, proving the need to consider asymptomatic intestinal carriage prior to corticosteroid administration as well. The reason that only a subset of people infected with amebiasis develop clinical disease is poorly understood and challenging to study, given the complexity of host-amebic interactions. Ultimately, it is the combination of amebic virulence factors and destructive host inflammatory responses that mediate tissue injury seen with amebic colitis.^47^ Cell-mediated responses appear to provide some protection against amebiasis [45]. For example, macrophages and neutrophils activated by cell mediated interferon-γ kill trophozoites in vitro [46,47]. In addition, children with higher levels of interferon-γ production have lower susceptibility to subsequent symptomatic amebiasis [48]. The development of fulminant amebic colitis in patients treated with corticosteroids further supports the importance of cell-mediated immunity in controlling *E*. *histolytica* infection. Rodent models of amebic colitis have demonstrated causal relationships between corticosteroids and exacerbation of amebic disease [49–51]. However the exact mechanism leading to such rapid and expansive intestinal inflammation and necrosis is unknown. That said, *E*. *histolytica* encodes for a macrophage migration inhibitory factor (MIF) homolog which has been shown to inhibit the anti-inflammatory responses of corticosteroids in vitro, potentially promoting a pro-inflammatory state [52].

Patients with amebic colitis may present initially with acute onset abdominal pain, diarrhea and bloody stools or less commonly with a more chronic course of diarrhea, weight loss and abdominal pain. Many of these symptoms overlap with those of inflammatory bowel disease, and even stool inflammatory markers, imaging, endoscopic findings and lesion distribution in amebic colitis can be non-distinguishing and difficult to differentiate from inflammatory bowel disease [53]. A number of diagnostic modalities, including stool studies, histology, and serology are available to assist, though a combination of techniques is often used to establish the presence of infection.

**Fig 2:**
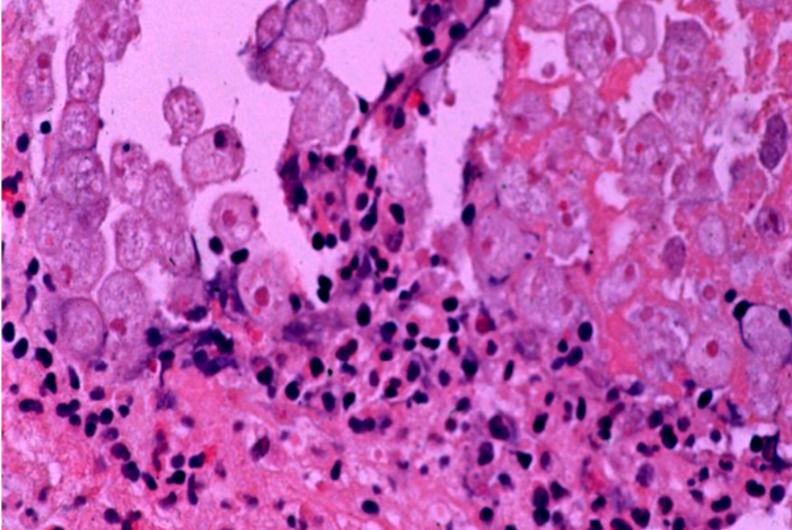
Invasion of colonic mucosa by amebic trophozoites with resultant inflammatory response. Colonic biopsy taken from a 49-year-old US male migrant from Mexico, diagnosed with severe amebic colitis after receiving high dose dexamethasone during management of subarachnoid hemorrhage (Courtesy of William A. Petri, Jr., University of Virginia)

Cysts and trophozoites (with or without hemophagocytosis) can be visualized by an experienced eye using stool microscopy, but this test lacks specificity. Advances in molecular epidemiology have revealed that three other *Entamoeba* species associated with human infection are morphologically indistinguishable from *E*. *histolytica* (including *E*. *dispar*, *E*. *moshokovskii* and *E*. *bangladeshi*) and cannot be differentiated from *E*. *histolytica* by microscopy [54]. In addition, stool excretion is variable and multiple stool samples must be submitted to maximize the chance of visualization by microscopy. At best, the sensitivity of stool microscopy is only 60%, consistent with the sensitivity of 50% noted in our study, yet this modality remains the most widely used test of diagnosis worldwide [55]. More sensitive methods are available and stool microscopy is no longer recommended for diagnosis. Several antigen detection tests specific for *E*. *histolytica* have been recently developed, such as the Techlab *E*. *HISTOLYTICA II* test (detects *E*. *histolytica*-derived Gal/ GalNAc-specific lectin), offering high sensitivity and specificity, up to 94% and 100% respectively [56–58]. Antigen detection tests are simple to perform, rapid, can be tested in batches, and may even be combined to simultaneously detect multiple parasitic enteropathogens, but unfortunately, despite these advantages, remain underutilized, as evidenced by only a single report in our study documenting use of a rapid antigen detection test [26,59]. Other tests, such as rapid detection of lectin antigen in stool and serum are under development [57]. Polymerase chain reaction (PCR) methods also show high sensitivity and specificity, but are mostly available as research tools, though may prove to be more useful as commercial tests in the future [60]. Detection of antibodies by serologic assay (indirect fluorescent, counter immunoelectrophoresis or enzyme linked immunosorbent assay) is often possible by the time of presentation. Serology may be a useful adjunct to stool studies. The sensitivity of serology ranges from 60–90%, consistent with the sensitivity of 67% found in our study. The utility of serology in endemic areas with high seroprevalence is limited, however, as serology may remain positive years after infection. In more severe cases, histology obtained by biopsy at colonoscopy (**Fig 2**), flexible sigmoidoscopy or surgical resection may show ulcers (e.g., classic flask-shaped ulceration), sometimes with cysts and trophozoites found at the lesion edge, and proved useful in 47% of the cases undergoing endoscopy and 89% of patients undergoing surgical resection in our cases studied.

All patients with evidence of *E*. *histolytica* infection should receive treatment, even if asymptomatic, to prevent progression, especially as the potential always exists for unknown future need to administer corticosteroids. For amebic colitis and other symptomatic forms of disease, a two-drug approach is taken with an amebicidal agent, such as the nitroimidazole metronidazole [61], to eliminate invading trophozoites and a luminal cysticidal agent such as the non-absorbable aminoglycoside paromomycin to eradicate intraluminal carriage of cysts [62]. If screening reveals asymptomatic carriage, then treatment with a luminal agent is sufficient to prevent progression of disease and transmission of infection [5]. Patients who are severely ill with amebic colitis should be hospitalized; supportive and intensive care should be provided as indicated. The addition of broad spectrum antibiotics may be required in the presence of peritonitis. Urgent surgical intervention is needed in the setting of bowel perforation. Toxic megacolon or extensive necrosis may require colectomy. In our study, almost half of cases required surgical intervention and a quarter died, despite appropriate antimicrobial therapy, fortifying the high morbidity and mortality associated with fulminant amebic colitis and the urgent need for improved therapeutic options. We did not find prior transplant, HIV infection, CMV infection, perforation, surgery or use of additional immune modulating therapies to be associated with the odds of death.

While there is no vaccine available to prevent amebiasis, the demonstration of at least partially protective host humoral and cell-mediated immune responses supports the need to continue efforts towards vaccine development [63]. In the meantime, travelers to endemic areas should be given advice on the avoidance of risk factors for acquisition of infection, and counselled on the use of proper hand hygiene, food and water precautions and avoidance of fecal exposure during sexual activity [64].

There are several limitations to our study. Severe and fulminant forms of colitis in patients treated with steroids are likely vastly underreported in the literature. Due to the retrospective nature and extraction of data from prior reports, incomplete information was collected from case descriptions. The small sample size may have limited the power to detect significant associations.

### Conclusions

Our study describes 24 cases of patients who developed severe and fulminant colitis following treatment with corticosteroid therapy, emphasizing the high morbidity and mortality associated with this condition and identifying knowledge gaps that must be addressed in the future. Efforts should be made to improve familiarity of this diagnosis among health care providers. In endemic countries, infection with *E*. *histolytica* must be excluded prior to the diagnosis of inflammatory bowel disease and treatment with corticosteroids administered systemically or by enema. Empiric treatment of amebiasis should be considered in situations where diagnostic testing is not readily available. In non-endemic countries, prior to starting corticosteroids or other immune suppressive therapy, travel history of the patient, close household and sexual contacts should be obtained. For those who have traveled to or migrated from endemic countries, screening for amebiasis should be carried out and those with positive test should be treated (**Table 2**). The most sensitive and specific test(s) available should be chosen to satisfactorily exclude. Consideration of *E*. *histolytica* in such situations will help to avoid a delay in diagnosis and potential death [65].

**Table 2:** Summary of findings and recommendations.

Patients with either symptomatic or asymptomatic intestinal amebiasis treated with corticosteroid therapy are at high risk of developing the potentially fatal complication of fulminant amebic colitis.
Infection with *E*. *histolytica* should be considered prior to the diagnosis of inflammatory bowel disease, and for subsequent exacerbations.
Travel history of patients, their close house hold and sexual contacts should be obtained prior to initiation of systemic corticosteroids. Patients residing in or with travel history to endemic parts of the world, such as South and Southeast Asia, Africa, Central America, South America and Mexico, should be screened for amebiasis with tests that afford the highest level of sensitivity and specificity available.
All patients with evidence of *E*. *histolytica* infection should be treated appropriately prior to initiating corticosteroids to prevent fulminant amebic colitis. Amebic colitis should be treated with metronidazole followed by a luminal agent, such as paromomycin. Treatment with a luminal agent alone is sufficient for patients with asymptomatic intestinal amebiasis.
Research efforts are needed to develop both interventions to prevent amebic colitis, and additional therapies to treat fulminant amebic colitis are needed to improve outcomes.

## Supporting Information

S1 Checklist STROBE Checklist. (DOCX)
