# Supplementary material for: Fulminant Amebic Colitis after Corticosteroid Therapy: A Systematic Review
Source: PLoS Negl Trop Dis. 2016 Jul 28;10(7):e0004879. doi: 10.1371/journal.pntd.0004879 (PMC4965027; doi:10.1371/journal.pntd.0004879)
Supplement: S1 Checklist — (DOCX) [file pntd.0004879.s001.docx]

STROBE Statement—checklist of items that should be included in reports of observational studies

|  | | | Item No. | | Recommendation | Page  No. | | Relevant text from manuscript |
| --- | --- | --- | --- | --- | --- | --- | --- | --- |
| Title and abstract | | | 1 | | (*a*) Indicate the study’s design with a commonly used term in the title or the abstract | 1 | | Title “Fulminant amebic colitis after corticosteroid therapy: a systematic review” |
|  |  |  |  |  | (*b*) Provide in the abstract an informative and balanced summary of what was done and what was found | 2 | | “We conducted a review of the English literature to describe cases of fulminant amebic colitis associated with exposure to corticosteroid medications, in order to identify the risk factors for poor outcome and determine difficulties in diagnosis and treatment. Articles reporting severe and fulminant forms of amebic colitis between 1991 and 2016 were collected. 525 records were screened to identify 24 cases for qualitative analysis associated with corticosteroid use.” |
| Introduction | | | | | | | |  |
| Background/rationale | | | 2 | | Explain the scientific background and rationale for the investigation being reported | 4 | | “Case reports have indicated that corticosteroids are a risk factor predisposing to the development of fulminant amebic colitis” |
| Objectives | | | 3 | | State specific objectives, including any prespecified hypotheses | 5 | | “Our aim was to systematically review recent articles reporting fulminant amebic colitis in patients treated with  corticosteroids to identify the main risk factors for poor outcome and highlight challenges regarding diagnosis and treatment.” |
| Methods | | | | | | | |  |
| Study design | | | 4 | | Present key elements of study design early in the paper | 5 | | “Systematic review of articles published in the English literature between  January 1991 and May 2016” |
| Setting | | | 5 | | Describe the setting, locations, and relevant dates, including periods of recruitment, exposure, follow-up, and data collection | 5 | | “The search was performed electronically in PubMed to find all articles reporting amebic colitis, using the following strategy: disease ("amoebic colitis"[All  Fields] OR "dysentery, amebic"[MeSH Terms] OR ("dysentery"[All Fields] AND "amebic"[All Fields]) OR "amebic dysentery"[All Fields] OR ("amebic"[All Fields] AND "colitis"[All Fields]) OR  "amebic colitis"[All Fields]) AND (("199101/01"[PDAT] "2016/05/01"[PDAT]) AND  "humans"[MeSH Terms]). The search was last conducted on May 5, 2016. A similar search was performed using Google Scholar to find additional articles that may not have been cataloged in  PubMed.” |
| Participants | | | 6 | | (*a*) *Cohort study*—Give the eligibility criteria, and the sources and methods of selection of participants. Describe methods of follow-up  *Case-control study*—Give the eligibility criteria, and the sources and methods of case ascertainment and control selection. Give the rationale for the choice of cases and controls  *Cross-sectional study*—Give the eligibility criteria, and the sources and methods of selection of participants | 5 | | “Case reports of intestinal amebiasis were then then reviewed to determine  corticosteroid exposure, including administration of systemic preparations (prednisone, prednisolone, methylprednisolone, triamcinolone, dexamethasone, cortisone acetate and  hydrocortisone) as well as enema preparations (budesonide). Evidence of amebiasis included identification by stool study, tissue examination or serology. Fulminant colitis was defined as  severe abdominal pain, dysentery, fever, peritonitis, perforation or the need for urgent surgical intervention” |
|  |  |  |  |  | (*b*) *Cohort study*—For matched studies, give matching criteria and number of exposed and unexposed  *Case-control study*—For matched studies, give matching criteria and the number of controls per case |  | | N/A |
| Variables | | | 7 | | Clearly define all outcomes, exposures, predictors, potential confounders, and effect modifiers. Give diagnostic criteria, if applicable | 8 | | Age, primary steroid indication, co-morbidity, steroid formulation, other immune modulating medications, country reported, risk factor for acquisition, symptoms, lab findings, area of gut involved, treatment, surgical intervention, survival. |
| Data sources/ measurement | | | 8* | | For each variable of interest, give sources of data and details of methods of assessment (measurement). Describe comparability of assessment methods if there is more than one group |  | | N/A |
| Bias | | | 9 | | Describe any efforts to address potential sources of bias | 5 | | “Systematic review of articles published in the English literature between January 1991 and May 2016” |
| Study size | | | 10 | | Explain how the study size was arrived at | 6 and 26 | | “Our search strategy identified 514 publications. Eleven additional publications were identified  using the alternative search engine, excluding duplicate publications. By evaluation of the title and abstract, 404 publications were excluded. Full text publications were then reviewed for eligibility including reports of intestinal amebiasis and concomitant administration of corticosteroid therapy. Among the 122 publications reviewed, a total of 23 publications, with 24 cases were found in the literature” |
| Quantitative variables | | 11 | | Explain how quantitative variables were handled in the analyses. If applicable, describe which groupings were chosen and why | | 5 | “odds ratios were calculated to measure association between  exposure and outcome” | |
| Statistical methods | | 12 | | (*a*) Describe all statistical methods, including those used to control for confounding | | 5 | “odds ratios were calculated to measure association between  exposure and outcome” | |
|  |  |  |  | (*b*) Describe any methods used to examine subgroups and interactions | |  | N/A | |
|  |  |  |  | (*c*) Explain how missing data were addressed | |  | N/A | |
|  |  |  |  | (*d*) *Cohort study*—If applicable, explain how loss to follow-up was addressed  *Case-control study*—If applicable, explain how matching of cases and controls was addressed  *Cross-sectional study*—If applicable, describe analytical methods taking account of sampling strategy | |  | N/A | |
|  |  |  |  | (*e*) Describe any sensitivity analyses | |  | N/A | |
| Results | | | | | | | | |
| Participants | | 13* | | (a) Report numbers of individuals at each stage of study—eg numbers potentially eligible, examined for eligibility, confirmed eligible, included in the study, completing follow-up, and analysed | | 6 and 26 | “Our search strategy identified 514 publications. Eleven additional publications were identified  using the alternative search engine, excluding duplicate publications. By evaluation of the title and abstract, 404 publications were excluded. Full text publications were then reviewed for  eligibility including reports of intestinal amebiasis and concomitant administration of corticosteroid therapy. Among the 122 publications reviewed, a total of 23 publications, with 24  cases were found in the literature” | |
|  |  |  |  | (b) Give reasons for non-participation at each stage | | 6 |  | |
|  |  |  |  | (c) Consider use of a flow diagram | | 26 |  | |
| Descriptive data | | 14* | | (a) Give characteristics of study participants (eg demographic, clinical, social) and information on exposures and potential confounders | | 8 |  | |
|  |  |  |  | (b) Indicate number of participants with missing data for each variable of interest | | 8 |  | |
|  |  |  |  | (c) *Cohort study*—Summarise follow-up time (eg, average and total amount) | |  | N/A | |
| Outcome data | | 15* | | *Cohort study*—Report numbers of outcome events or summary measures over time | | 12 | “Eleven (46%) underwent surgical treatment of their disease (**Table 1**). There were 6 fatalities (25%), half underwent surgical intervention prior to death” | |
|  |  |  |  | *Case-control study—*Report numbers in each exposure category, or summary measures of exposure | |  |  | |
|  |  |  |  | *Cross-sectional study—*Report numbers of outcome events or summary measures | |  |  | |
| Main results | | 16 | | (*a*) Give unadjusted estimates and, if applicable, confounder-adjusted estimates and their precision (eg, 95% confidence interval). Make clear which confounders were adjusted for and why they were included | |  | N/A | |
|  |  |  |  | (*b*) Report category boundaries when continuous variables were categorized | |  | N/A | |
|  |  |  |  | (*c*) If relevant, consider translating estimates of relative risk into absolute risk for a meaningful time period | |  | N/A | |
| Other analyses | 17 | | | Report other analyses done—eg analyses of subgroups and interactions, and sensitivity analyses | |  | N/A | |
| Discussion | | | | | | | | |
| Key results | 18 | | | Summarise key results with reference to study objectives | | 17 | “Our study describes 24 cases of patients who developed severe and fulminant colitis following treatment with corticosteroid therapy, emphasizing the high morbidity and mortality associated with this condition and identifying knowledge gaps that must be addressed in the future.” | |
| Limitations | 19 | | | Discuss limitations of the study, taking into account sources of potential bias or imprecision. Discuss both direction and magnitude of any potential bias | | 17 | “There are several limitations to our study. Severe and fulminant forms of colitis in patients treated with steroids are likely vastly underreported in the literature. Due to the retrospective nature and extraction of data from prior reports, incomplete information was collected from case descriptions. The small sample size may have limited the power to detect significant associations.” | |
| Interpretation | 20 | | | Give a cautious overall interpretation of results considering objectives, limitations, multiplicity of analyses, results from similar studies, and other relevant evidence | | 19 | Table 2 | |
| Generalisability | 21 | | | Discuss the generalisability (external validity) of the study results | | 19 | Table 2 | |
| Other information | | | |  | | | | |
| Funding | 22 | | | Give the source of funding and the role of the funders for the present study and, if applicable, for the original study on which the present article is based | |  | NIH grant R01 AI026649  NIH grant K08AI119181 | |

*Give information separately for cases and controls in case-control studies and, if applicable, for exposed and unexposed groups in cohort and cross-sectional studies.

**Note:** An Explanation and Elaboration article discusses each checklist item and gives methodological background and published examples of transparent reporting. The STROBE checklist is best used in conjunction with this article (freely available on the Web sites of PLoS Medicine at http://www.plosmedicine.org/, Annals of Internal Medicine at http://www.annals.org/, and Epidemiology at http://www.epidem.com/). Information on the STROBE Initiative is available at www.strobe-statement.org.
